# Inflammatory mechanisms underlying cortical injury in progressive multiple sclerosis

**DOI:** 10.20517/2347-8659.2020.35

**Published:** 2021-06-20

**Authors:** Leah R. Zuroff, Joyce A. Benjamins, Amit Bar-Or, Robert P. Lisak

**Affiliations:** 1 Perelman School of Medicine, University of Pennsylvania, Philadelphia, PA 19104, USA.; 2 Departments of Neurology and Biochemistry, Microbiology, and Immunology, Wayne State University School of Medicine, Detroit, MI 48201, USA.; 3 Neurology Department and Center for Neuroinflammation and Experimental Therapeutics, Perelman School of Medicine, University of Pennsylvania, Philadelphia, PA 19104, USA.

**Keywords:** Progressive multiple sclerosis, cortical lesions, subpial cortical demyelination, meningeal inflammation

## Abstract

Multiple sclerosis (MS) is a lifelong inflammatory demyelinating disease of the central nervous system (CNS). While there has been substantial progress in the development of therapeutic strategies for relapsing disease, the field has lagged behind in its understanding and management of progressive stages of the disease, including secondary progressive and primary progressive MS, respectively. It is now thought that distinct but temporally overlapping mechanisms underlie relapsing and progressive aspects of the disease. Relapsing disease is characterized by waves of peripheral immune cell activation and CNS infiltration leading to focal destruction of the white matter, while progressive disease is thought to be driven by chronic, low-grade multifocal inflammation contained within the CNS compartment. Specifically, peripheral B cells, T cells, and myeloid cells take up residence within niches of the inflamed CNS, such as the leptomeninges and the Virchow-Robin spaces, where complex interactions between peripheral and CNS resident cells serve to maintain these cellular aggregates and further propagate CNS injury. In particular, immune infiltrates within the meninges are tightly associated with a specific form of cortical injury, termed subpial cortical demyelination, which is thought to be a key pathologic driver of disease progression. Cortical injury in the MS brain likely occurs via a combination of multiple immune-mediated and degenerative processes, perhaps including the production of diffusible toxic mediators by peripheral immune cells retained within the meninges. A better understanding of the interplay between peripheral immune and CNS resident cells is not only relevant to our concept of the disease process, but also represents a novel target for therapeutic intervention that is more specific to progressive disease biology. This review will focus on the role of CNS-compartmentalized inflammation in the development of cortical injury in MS, with a particular emphasis on the importance of immune-CNS crosstalk in disease progression.

## INTRODUCTION

Multiple sclerosis (MS) is a chronic inflammatory and degenerative demyelinating disease of the central nervous system (CNS) with variable clinical course and rates of progression of neurological disability. Classical teaching has distinguished between relapsing-remitting MS (RRMS) and progressive forms of MS, which in 10%−15% of patients manifests clinically from disease onset [primary progressive MS (PPMS)] or, in most patients, emerges after initial onset with relapsing-remitting disease [secondary progressive MS (SPMS)]^[1–4]^. Mounting evidence from neuropathological, clinical, and imaging studies now support the view that these clinical labels do not define temporally distinct biological entities. Indeed, individuals throughout the MS spectrum can experience both acute focal inflammatory injury (reflecting relapse biology) and the more insidious and distributed progressive injury (reflecting non-relapsing, progressive biology)^[4,5]^. In addition to their substantial temporal overlap, both of these processes contribute to subclinical CNS injury^[6–8]^. It follows that MS is now thought of more as a continuum, from radiologically isolated syndrome (RIS), through clinically isolated syndrome (CIS), RRMS, and SPMS, with PPMS differing from SPMS only by the lack of clear-cut clinically evident relapses^[4]^.

While accomplishments of the last several decades are highlighted by the development of increasingly effective disease-modifying therapies (DMTs)^[2,9]^, treating progressive disease remains a major outstanding challenge. Only three DMTs, ocrelizumab, siponimod, and cladribine, have been approved for the treatment of patients with progressive forms of MS, all showing relatively modest benefit on progression^[10–14]^. The question remains to what extent this benefit indeed reflects targeting non-relapsing progressive disease (i.e., clinical worsening in the absence of clinical or radiographic relapses, sometimes referred to as progression independent of relapse activity, or PIRA)^[5]^, as opposed to progression due to relapses that do not fully recover. In general, even the immunotherapies that are highly effective in targeting the pathophysiological mechanisms underlying relapse biology in MS have a rather limited capacity to impact the temporally overlapping but distinct process(es) that drive(s) non-relapsing disease progression^[10–13]^.

A wealth of neuropathological and imaging data demonstrates the changing features of inflammation and neural tissue degeneration across the spectrum of MS^[10–12,14–16]^. It is thought that relapses result from waves of peripheral immune cell activation and focal perivascular infiltration into the CNS across a disrupted blood-brain barrier (BBB), resulting in acute inflammatory demyelination and axonal injury^[17–19]^. Meanwhile, progressive disease biology involves chronic, low-grade inflammation that is thought to foster immune cell retention within the CNS. The resulting CNS-compartmentalized inflammation then propagates ongoing demyelination and neuronal loss without relying on the episodic recruitment of peripheral immune cells into the CNS^[18,20–22]^. Over time, the reduction in prominent relapse biology and the associated decrease in development of new gadolinium-enhancing lesions on MRI means that the BBB may be less permeable in patients with predominantly progressive disease, which in turn may both foster CNS-compartmentalized inflammation and limit penetration of DMTs^[18,22]^. Even for the group of small molecule DMTs that do penetrate the CNS^[23–25]^, evidence for a direct effect on CNS protection and/or repair in humans is lacking^[26]^.

With the changing character of inflammation through the disease course, there is also a shift in key pathological findings. While acute, focal, demyelinating plaques predominate early in RRMS, their relative contribution to total white matter lesion (WML) burden wanes in later stages of the disease^[10,14,21,27]^. The white matter pathology now thought to contribute to progressive MS involves a subset of perivascular lesions with a lower but persistent degree of inflammation, which pathologically have been considered “chronic active” lesions^[10,14]^ and by imaging may be captured as the more recently described slowly evolving lesions (SELs) or “rim” lesions^[14,21,28–31]^. Other pathological changes also become more prominent with advancing disease, such as demyelination and degeneration of both the superficial and deep gray matter structures as well as more subtle and distributed inflammatory changes in the normal-appearing white and gray matter^[10,13,16,32–34]^. Though each of these pathological findings is thought to contribute to disease progression in some capacity^[32,34–36]^, a growing body of literature suggests that cortical injury is one of the strongest predictors of progression, including both progression of physical disability and cognitive decline^[35–38]^ as well as evolution of the disease course from initial clinical episodes to SPMS^[38–40]^. A summary of studies linking cortical pathology and disease progression in MS is provided in Table 1 and builds on the table provided in the detailed review by Geurts *et al*.^[35]^ in 2012. Though cortical lesions are described as more numerous and more extensive in the brains of progressive MS patients examined at autopsy^[16]^, they are also reported in significant proportions of patients early in their course of relapsing disease^[41,42]^, including CIS^[39]^ and RIS^[43]^. Because these studies in early-stage MS patients utilized MRI rather than histopathology to detect cortical lesions, the true prevalence of cortical pathology was likely substantially underestimated. This further supports the notion that relapsing and progressive disease biology occurs simultaneously in the MS CNS.

This review will describe the major patterns of cortical injury (leukocortical, intracortical, and subpial lesions) and their relative contribution to disease progression. As will be described in greater detail below, leukocortical/intracortical lesions are thought to reflect mechanisms of perivascular inflammatory injury that are likely shared with classic WMLs. In contrast, the subpial component of cortical injury is thought to reflect progressive, non-relapsing disease biology^[44,45]^, which is the focus of this review. We will therefore emphasize those studies of potential mechanisms underlying subpial lesion formation and their relevance to disease progression. The key word search for this review included variations in the following terms between 1990 and 2020 in the English language: cortical lesions/injury/demyelination, subpial lesions/injury/demyelination, progressive multiple sclerosis, and multiple sclerosis disability progression. The overarching goal of the discussion is to emphasize key pathways that may represent meaningful therapeutic targets in progressive disease, while also recognizing some of the knowledge gaps and technical limitations that must be addressed to ultimately develop and translate such approaches. Given our focus on cellular and molecular mechanisms of cortical injury, and subpial cortical injury in particular, we limit our commentary on imaging to a selection of *in vivo* studies that provide complementary insights to particular pathophysiological considerations, and we refer the readers to recent comprehensive reviews on imaging of cortical lesions in MS^[46–48]^.

## PATTERNS OF CORTICAL INJURY IN MULTIPLE SCLEROSIS

**Figure 1.**
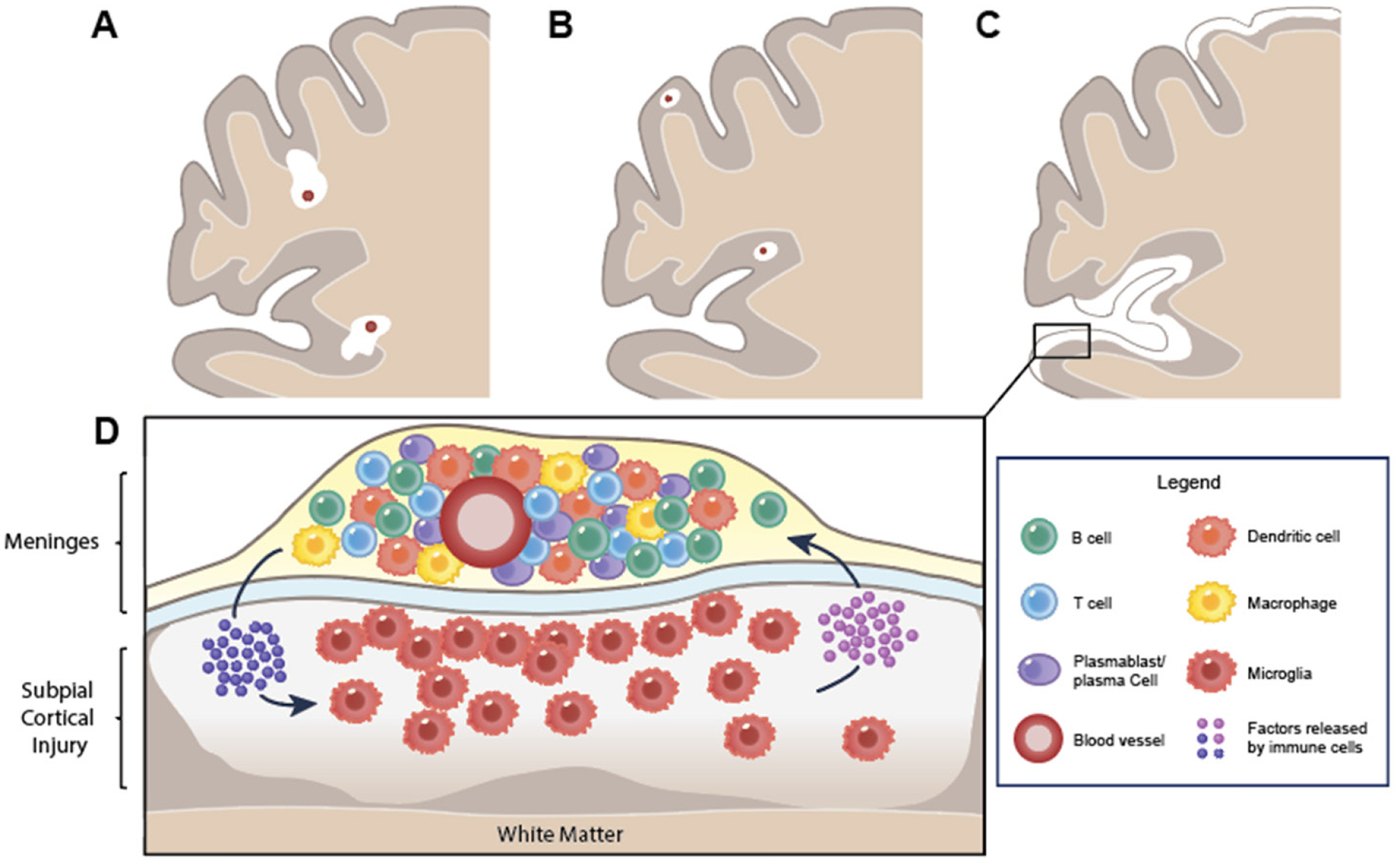
Cortical lesions in multiple sclerosis. Coronal brain sections demonstrating cortical demyelination (white) are shown in panels A-C. (A) Leukocortical (type I) and (B) intracortical (type II) lesions form around post-capillary venules (red) in the subcortical white and cortical gray matter, respectively; (C) subpial (type III) lesions extend from the superficial pial surface into the deeper layers of cortex. Type IV lesions involve all cortical layers and are not shown specifically in this schematic; (D) magnified representation of subpial cortical lesion segment demonstrating proposed inflammatory mechanism underlying subpial injury in MS. Meningeal immune cell aggregates are tightly associated with subpial lesions, in which a gradient of demyelination and microglial activation in the subjacent cortex is represented. Meningeal immune cell aggregates are typically B cell-rich and contain proliferating CD20+ B cells, plasmablasts/plasma cells, and CD4+ and CD8+ T cells contained within a network of follicular dendritic cells (see Legend). Mediators released by infiltrating immune cells in the meninges as well as by CNS-resident cells, such as activated microglia and astrocytes, are thought to contribute to ongoing meningeal inflammation and subpial injury in multiple sclerosis. A range of mechanisms have been implicated as underlying subpial cortical injury, including (1) secretion of diffusible toxic factor(s) (e.g., pro-inflammatory cytokines, extracellular vesicles, complement, *etc*.) released by immune cells in the meninges and/or perivascular spaces; (2) pro-inflammatory and/or toxic substances (e.g., oxygen radicals; nitrogen species; excitotoxic glutamate) released or insufficiently cleared by activated and injured glial cells; (3) mitochondrial dysfunction leading to bioenergetic failure within neurons and oligodendrocytes; and (4) neural-glial uncoupling (including loss of trophic support). CNS: central nervous system; MS: multiple sclerosis

Four types of demyelinated lesions have been described in the cortex of MS patients: leukocortical (type I), intracortical (type II), and subpial (type III, sometimes further divided into types III and IV)^[49–51]^. Leukocortical lesions are thought to originate as a perivascular lesion in the white matter that extends across the gray-white matter junction to involve the cortex. Intracortical lesions reside entirely within the cortex, are typically small, and form in a perivascular distribution, similar to type I lesions and focal lesions in the white matter. In contrast, subpial cortical lesions extend from the pial surface (layer I of the cortex) into the deeper cortical layers. Most lesions involve superficial cortical layer I through layers III or IV (referred to as type III subpial cortical lesions), while some involve the entire thickness of the cortex (type IV subpial cortical lesions), notably with remarkable respect for the gray-white matter junction. The varied topography of these lesions suggests that different mechanisms may drive their development and maintenance [Figure 1], and that different cortical lesion types may contribute to progressive biology and disease progression to varying extents. That being said, certain features distinguish cortical lesions in general from lesions in the white matter, namely that they are typically much less inflammatory, as measured by the number of parenchymal and perivascular infiltrates^[49,50]^, and interestingly, they seem to possess a greater capacity for remyelination, perhaps related to the greater number of oligodendrocyte precursor cells observed in the demyelinated cortex^[52–54]^.

Prior to discussing the potential mechanisms underlying the development and clinical consequence of these different cortical lesions, there are several challenges to studying cortical lesions in general that should be considered. First, correlating clinical outcomes with pathological findings requires tissue samples that are generally obtained via either post-mortem autopsy or by clinically indicated stereotactic brain biopsy. In either case, the individuals studied may not be representative of the general MS population. Post-mortem tissue typically originates from patients at the latest stages of disease and may fail to capture key mechanisms involved in prior tissue injury. In turn, biopsy specimens are obtained from a group of individuals with enough atypical lesional activity to warrant tissue sampling^[16,41,55]^. That being said, some post-mortem studies do include patients who have died earlier in their disease course, and cortical lesions with varying degrees of inflammatory activity have been reported even in later stages^[44,56–58]^. *In vivo* assessment, including prospective monitoring, of cortical lesions has relied on MRI, though most early studies found that identification of cortical lesions using 3T MRI was largely restricted to the leukocortical lesions. The introduction of ultra-high-field 7T MRI and more sensitive imaging sequences, such as double-inversion recovery^[59]^, phase-sensitive inversion recovery^[59,60]^, magnetization transfer imaging^[61]^, diffusion-weighted imaging^[62]^, and 3D T1-weighted magnetization-prepared rapid gradient echo^[63,64]^, have improved cortical lesion resolution, though capturing all cortical lesions *in vivo* remains challenging. This is likely due to their small size (particularly the intracortical lesions, which are also less common), the absence of significant surrounding edema, and proximity to the CSF in the case of subpial lesions^[35]^. For instance, in one post-mortem verification study of cortical lesions, 7T MRI detected 100% of leukocortical lesions identified pathologically, but only 36.2% of intracortical and 13.6% of subpial lesions^[65]^. Until new or improved imaging modalities enable more complete detection of intracortical and subpial lesions^[35,60,63–67]^, current MRI-based assessment of these lesions may underestimate their association with clinical outcomes. Recent improvement in PET imaging modalities, such as with (11) C-PBR28 MR-PET^[34,68]^, which can detect activated myeloid cells, may provide a window into the relevant sites and pathophysiological mechanisms involved in progressive disease. Further validation is greatly needed to both fully evaluate the respective contributions of cortical lesions to disease progression and to better evaluate treatment effects on distinct mechanisms underlying cortical pathology.

### Leukocortical lesions

Leukocortical lesions are demyelinating plaques that involve the subcortical white matter and adjacent cortex. Pathologically, leukocortical lesions appear to originate around a central vein or venule in the subcortical white matter and spread out radially to involve part of the overlying cortex^[50,51]^. They are generally more inflammatory than the other cortical lesions^[49,50]^ and are strongly associated with features of adjacent WMLs^[15,69,70]^. Indeed, the inflammatory activity in leukocortical lesions seems to evolve in a similar pattern to that documented in WMLs^[55,57]^, and the degree of oxidative injury and neuronal loss can be explained, at least in part, by retrograde degeneration from injured axons in the white matter^[15]^. For these reasons, it is thought that leukocortical lesions and WMLs may form via similar mechanisms.

The frequency of leukocortical lesions in MS brains largely depends on the population studied. Leukocortical lesions were shown to represent anywhere from 15% to 34% of the total cortical lesion burden in post-mortem tissue^[49,51]^ or up to 50% in biopsy specimens obtained from early stage MS patients^[41]^. Because the formation of active WMLs occurs more frequently in early MS^[14]^, this may explain the higher proportion of leukocortical lesions observed in this phase of disease.

A few studies have investigated the relative contribution of different cortical lesions to disease outcomes, and it is interesting to note that several have demonstrated a robust association between leukocortical lesions and cognitive impairment even at early phases of disease^[69,70]^. The particular effect on cognition may be due to disruption of subcortical U-fibers and, consequently, cortical-cortical signaling, which has previously been implicated in cognitive impairment^[69–71]^.

The presumed white matter origin of leukocortical lesions (as focal perivascular inflammatory plaques) is thought to be driven, like other WMLs, by CNS infiltration of autoreactive T cells, which are subsequently reactivated in the meninges, CSF, or perivascular spaces^[9,72,73]^. This is thought to trigger a second wave of inflammation and infiltration around a central vein or venule, consisting mainly of CD8+ and CD4+ T cells, B cells, and monocytes^[12,74,75]^. CD8+ T cells and macrophages may then infiltrate the surrounding parenchyma, and this massive influx of immune cells leads to pronounced demyelination, axonal injury, and further breakdown of the BBB^[17–19]^. Indeed, contrast-enhancing leukocortical lesions were identified in over one-third of early MS patients, indicating significant BBB disruption is involved in their development^[76]^. Over time, inflammatory activity within the lesion core subsides, and they may be classified as either mixed active/inactive or inactive lesions depending on the degree of demyelination and inflammation^[77]^. Some active/inactive WMLs may manifest as SELs at sites of prior perivascular inflammatory demyelinating lesions and are now thought to contribute pathophysiologically to non-relapsing disease progression^[14,21,28–31]^. The extent to which leukocortical or intracortical lesions may develop into SELs as described for WMLs is not known.

Though the full spectrum of active, mixed active-inactive, and inactive lesions has been described in the cortex of MS patients^[57]^, several studies suggest that the gray matter aspect of leukocortical lesions behaves somewhat differently from its white matter counterpart. For instance, the degree of inflammation (in terms of immune cell infiltrates) is always substantially less in the cortical component of these lesions than in their white matter component^[49,50]^. This manifests as reduced numbers of parenchymal T cells and activated CD68+ myeloid cells as well as nearly absent perivascular cuffs in post-mortem tissue samples^[49]^, though more significant inflammation can be seen in early MS^[41]^. The reason for this stark difference is not fully understood. It is possible that different phenotypes of CNS-resident immune cells, such as microglia and astrocytes, exist in these two tissues^[49,78–80]^. Though these CNS cells may not influence initial lesion formation, distinct glial phenotypes may respond differently to the initial demyelinating injury and, consequently, impact the composition and propagation of ongoing inflammation and damage^[49,78–80]^. Such potential differences between white and gray matter are worthy of further investigation, as they may ultimately be relevant to the effects of DMTs on various types of cortical pathology.

### Intracortical lesions

Intracortical lesions are similar to both leukocortical and typical WMLs in that they also form around post-capillary veins or venules, though they tend to be much smaller and, like the cortical components of leukocortical lesions, less inflammatory^[41,51]^. Differences in BBB permeability characteristics between the cortex and white matter may explain, at least in part, the reduced inflammatory activity observed in these cortical lesions^[18,22]^. Meanwhile, their perivascular distribution suggests that they too form via peripheral immune cell activation and infiltration of the brain parenchyma^[41,49,51,81]^ and, indeed, up to 23% of intracortical lesions in biopsy specimens were considered actively demyelinating, with the majority demonstrating both parenchymal and perivascular CD3+ and CD8+ T cells^[41]^. In autopsy tissue, the extent of intracortical lesions correlated with total WML load and the proportion of mixed active/inactive lesions, as well as with the presence of clusters of activated microglia/macrophages in normal-appearing white and gray matter^[36]^. Taken together, intracortical lesions have been associated with pathological features that may have elements of both relapse and progressive disease biology, and it remains to be determined whether the development and/or maintenance of these lesions are more closely associated with one or both of these processes. As for the leukocortical lesions noted above, whether and to what extent intracortical lesions may become SELs is unknown.

BBB permeability is an important feature of focal demyelination in the MS CNS. Recently, it has been shown that vascular leakage and deposition of fibrinogen, a serum protein involved in the clotting cascade, may actually precede and trigger neuroinflammation and demyelination in the white matter, and perhaps also the cortex^[82–84]^. In both rodent and primate models, fibrinogen was shown to activate local microglia in a CD11b-dependent manner, which subsequently triggered recruitment and expansion of myelin-specific Th1 cells^[82–84]^. Fibrinogen has been detected at the rim of chronic active lesions, further implicating an association with ongoing inflammation and demyelination^[83]^. With the genesis of both leukocortical and intracortical lesions thought to be surrounding a central vein, it is conceivable that fibrinogen plays a similar pathogenic role in the development of cortical pathology, as well. Indeed, more pronounced fibrinogen deposition has been described in the cortex of progressive MS patients compared to controls and was associated with reduced axonal density, specifically in the deeper layers of the cortex^[85]^. Additionally, elevated levels of fibrinogen in the CSF were associated with markers of innate immune inflammation and greater cortical lesion burden detected by MRI in recently diagnosed MS patients^[86]^, which together suggests a role for fibrinogen in both cortical demyelination and neurodegeneration^[87]^.

Further mechanistic evidence can be extrapolated from rodent models that recapitulate certain features of inflammatory cortical demyelination. In one recent study using stereotactic injections of IFNg and TNFa into the motor cortex of recombinant MOG_1–125_-immunized Th/+ mice, it was found that intracortical lesion formation is highly dependent on infiltration of encephalitogenic T cells, NK cells, and CCR2+ monocytes, all of which were also demonstrated in post-mortem demyelinated cortex^[55]^. While inflammatory macrophages were implicated in the development of both intracortical and subpial lesions in this model, the involvement of T cells and NK cells were only critical for intracortical lesion formation. In fact, the presence of encephalitogenic T cells was necessary to permit NK cell passage into the brain parenchyma of these mice^[55]^. While murine models are limited in their generalizability, particularly when it comes to progressive MS disease mechanism, these findings support the notion that distinct immune effector mechanisms may be responsible for the formation of different cortical lesion types.

### Subpial cortical lesions

Subpial lesions are the most common cortical lesion identified in post-mortem tissue, with varying prevalence and inflammatory activity across the disease spectrum. They are present in almost all cases of progressive disease and represent 50%−90% of the cortical lesion burden^[49,51,88,89]^. In biopsy material from patients with early RRMS or tissue blocks from RRMS of short duration, subpial lesions can represent anywhere from 34% to 62% of all cortical lesions^[41,88]^. In some cases, subpial cortical demyelination may even occur in the absence of WMLs^[90]^ or represent the dominant lesion type in the CNS^[91]^. The large range in lesion prevalence observed across studies may be due to the methods by which the specimens were obtained (post-mortem tissue vs. stereotactic brain biopsy) or sectioned and stained^[89]^, as well as the inherent heterogeneity of disease activity observed across the disease spectrum^[56,92]^.

Pathologically, subpial cortical lesions are distinct from other demyelinating plaques in that they do not form around a central vein and are relatively devoid of parenchymal and perivascular infiltrates^[15,50]^. Indeed, multiple studies have demonstrated that these lesions form independently of any vascular territory^[15,50]^. Instead, the demyelination extends from the upper cortical layer (most superficial cortical layer I) to involve several or occasionally all cortical layers, with lesions commonly involving multiple adjacent gyri^[15,44,45,49,51,93]^. A seminal study by Magliozzi *et al*.^[45]^ revealed that immune cell collections in the meninges (some with follicle-like features) are associated with areas of adjacent subpial cortical demyelination. The finding that subpial lesions exhibit a surface-in gradient of neuronal injury and microglial activation suggests that one or more toxic soluble factor(s) may be released by immune cells in the meninges and diffuse across the CSF to damage the glia limitans and underlying cortex^[44,45,93–95]^. Indeed, subpial cortical lesions and adjacent immune-cell meningeal infiltrates have a predilection for the deep invaginations of the cortex, including the cingulate and insular cortices, where CSF stasis may allow more prolonged exposure to such putative toxic molecules^[45,94]^.

It has consistently been shown that patients with a greater degree of meningeal inflammation and more pronounced subpial cortical pathology also have a more rapid and severe clinical course, characterized by younger ages at disease onset, wheelchair dependence, and death^[44,57,93,94]^, with the extent of subpial cortical lesions also correlating with both physical and cognitive disability^[70,96]^. Taken together, these findings raise several potentially clinically relevant questions: when and how are meningeal immune cell collections formed; how are they maintained in the MS CNS; and what are they releasing that may mediate damage to the subjacent cortex. All these questions are discussed in greater detail below.

An additional intriguing line of investigation pertains to the specificity of this pathological finding (and the presumed associated mechanism of injury) to MS. Subpial lesions were previously considered unique to MS, as they had not observed in a range of other neuroinflammatory or neurodegenerative conditions^[21,97,98]^ until the recent report describing their presence in autopsy and biopsy specimens from patients with myelin oligodendrocyte glycoprotein (MOG) antibody-associated disorders (MOGAD)^[99]^. Whether the features of cortical injury in MOGAD are identical to those seen in MS remains to be fully elucidated. This initial neuropathological investigation in MOGAD indicated that different cortical lesion subtypes predominate in the two conditions (intracortical in MOGAD *vs*. leukocortical and subpial in MS), though it should be noted that all but two MOGAD samples in this cohort were obtained via biopsy, which may influence estimates of the relative frequency and activity of cortical lesion subtypes observed^[99]^. The authors did not comment on whether the subpial lesions in MOGAD exhibited a surface-in gradient of inflammation and injury or whether their presence was associated with overlying meningeal immune-cell collections. Further comparative studies of cortical pathology and meningeal inflammation in MS and MOGAD will likely help elucidate important pathogenic mechanisms, both shared and unique, in these two disorders.

#### Mechanisms promoting initiation and maintenance of meningeal inflammation

Some degree of meningeal inflammation is now recognized to be a common feature in the MS CNS through all stages of disease, and the more prominent immune cell collections have been associated with greater underlying damage in the cerebral^[44,45,88,93,94]^ and cerebellar^[58]^ cortices as well as in the spinal cord^[100]^. It should be recognized that methodological differences in tissue preservation, sectioning, and staining as well as differences in disease presentation and phenotype, may all contribute to the differing estimates of the extent and profiles of meningeal immune cell aggregates in MS^[13,101,102]^. More diffuse (i.e., less organized) meningeal inflammation generally comprises CD20+ B cells and plasma cells/plasmablasts as well as T cells and activated macrophages^[44,45,95,103]^. A distinction should be made between diffuse meningeal inflammation and B cell-rich follicle-like structures, which are less common, smaller, and more highly structured aggregates. Though the more organized meningeal inflammation in MS can recapitulate some features of lymphoid follicles (hence referred to by some as follicle-like structures), they relatively infrequently recapitulate all features that would qualify them formally as tertiary lymphoid tissue. They are often B cell rich, comprising proliferating CD20+ B cells, plasma cells, and CD4+ and CD8+ T cells in a network of CD35+ follicular dendritic cells^[44,94,95]^. The more organized meningeal infiltrates have been identified in a subset of patients with both relapsing and progressive clinical phenotypes of MS, who seem to have a more severe clinical course^[44,45,57,94,95]^.

Evidence from both MS patients and experimental autoimmune encephalomyelitis (EAE) models has implicated pro-inflammatory cytokines and lymphocyte chemoattractants in the formation and maintenance of immune cell aggregates in the meninges^[104]^. Specifically, molecules promoting B cell recruitment and survival, such as CXCL13 and B cell activating factor (BAFF), are expressed by dendritic cells in the meninges^[44,95]^ and by infiltrating lymphocytes and astrocytes within active MS^[105,106]^ and EAE lesions^[107]^. Increased levels of BAFF and CXCL13 have also been reported in the CSF of patients with MS, particularly in SPMS patients with superimposed relapses or in RRMS patients during relapse^[108]^. Additionally, TNFa and IFNg^[55,96,109–112]^ as well as Th17^[111,113,114]^ signaling have been consistently implicated in the formation and maintenance of immune cell aggregates in EAE and potentially MS. Expanding on these findings, a recent study identified increased expression of the pro-inflammatory cytokines IFNg, TNFa, IL-2, and IL-22, as well as the molecules CXCL13, CXCL10, LTa, IL-6, and IL-10 in the meninges and CSF of SPMS cases with more pronounced meningeal inflammation and cortical pathology at time of death^[112]^. Similar patterns of inflammation were then demonstrated in the CSF from two independent cohorts of RRMS patients with more significant cortical injury at diagnosis, suggesting that these key inflammatory mediators may be important for the development and propagation of meningeal inflammation and potentially also cortical damage across all phases of disease^[112]^. The cellular source(s) of the abnormally increased levels of pro-inflammatory cytokines in the CSF of MS, and whether they are produced within the inflamed CNS or in the meningeal compartment, remain unknown.

As with *in vivo* monitoring of cortical lesions, understanding the natural history and evolution of meningeal inflammation in the MS CNS is greatly limited by the availability of an appropriate biomarker. Identifying foci of leptomeningeal contrast enhancement (LME) on MRI has been studied as a potential proxy for meningeal inflammatory aggregates, albeit with some limitations and conflicting results^[115–117]^. In one prospective imaging study, two progressive MS patients followed ante-mortem ultimately presented for autopsy, where it was possible to correlate *in vivo* imaging data with post-mortem 7T MRI and immunohistochemistry^[118]^. In these patients, MRI foci of LME present antemortem correlated with regions of meningeal T cell, B cell, and macrophage aggregates identified in the post-mortem tissue^[118]^. Of note, these meningeal aggregates were associated with regions of more pronounced cortical demyelination. Several other studies have since demonstrated an association between foci of LME *in vivo* and more pronounced cortical atrophy^[115,117,119]^, while others have failed to show an association^[116]^. It is important to note that the described structures with LME, which are visible to the eye, are of course orders of magnitude larger than microscopic aggregates of meningeal inflammation. The frequency of detection of LME is also relatively low compared to the relatively common meningeal immune cell aggregates, suggesting that if LME indeed represents actual sites of immune cell aggregates, they may be identifying relatively atypical ones, or just “the tip of the iceberg”. Additionally, LME is not specific to MS and has been described in other neuroinflammatory conditions^[120]^. Further study of LME and/or development of a separate imaging biomarker are greatly needed to better characterize and evaluate treatment effects on meningeal immune cell aggregates, which may play key pathophysiological roles in progressive disease mechanisms throughout the disease course.

#### Mechanisms by which meningeal immune cell aggregates may drive subpial cortical injury

Absence of direct contact between meningeal immune cells and the subjacent demyelinating cortex has drawn attention to the potential for soluble factors released by cells in the meninges to mediate injury to underlying cortical structures both directly and indirectly. For example, soluble factors released by meningeal immune cells could influence the transcriptional profile of underlying neurons and oligodendrocytes in such a way that increases their susceptibility to injury. A recent study using archival MS tissue specimens demonstrated that meningeal inflammation was associated with selective loss of *CUX2*-expressing excitatory neurons in the upper layers of the cortex, while other nearby excitatory and inhibitory neurons were relatively spared^[80]^. These vulnerable neurons upregulated genes for self-antigen presentation, including HLA-C and b-2 microglobulin, which may have been responsible for their increased susceptibility to injury. Similarly, another recent study in post-mortem SPMS tissue demonstrated that cortical neurons and oligodendrocytes in the brains of those with more pronounced meningeal inflammation, compared to those without, were found to exhibit increased expression of genes implicated in pro-apoptotic and pro-necroptotic signaling vs. pro-survival pathways^[92]^. The finding that many of these differentially expressed genes are regulated by TNF receptor signaling suggests that perhaps the presence of meningeal immune aggregates increases the sensitivity of cortical neurons and oligodendrocytes to TNF-mediated cell death^[92]^. Indeed, continuous expression of TNFa and IFNg in the meningeal compartment of MOG-immunized DA rats, delivered via lentiviral transfer vectors, recapitulated many aspects of the relationship between meningeal inflammation, subpial demyelination, and neuronal loss (including the upregulation of necroptotic pathways) observed in the post-mortem tissue of MS patients^[121]^. Further support comes from *in vitro* experiments in which rat primary neurons exposed to combinations of pro-inflammatory Th1- and monocyte/macrophage-derived cytokines exhibited altered gene expression in key pathways involved in neuronal health and apoptosis^[122]^. The fact that many rodent models of inflammatory cortical injury often incorporate injection^[55,109,110,123]^ or targeted delivery^[121,124]^ of TNFa and IFNg into the CSF or cortex indicates that these cytokines may be directly responsible for the cortical damage observed in these models, and perhaps also in MS patients, especially if a subset of cells exhibit increased susceptibility to cell death, as described above^[80,92]^. That being said, the introduction of these cytokines into the CNS compartment is also associated with meningeal inflammation in these models, and the observed pathology may be attributable to indirect effects on other cells^[125,126]^.

Beyond traditionally implicated molecules, a series of studies have demonstrated that products released into the medium by cultured MS patient-derived B cells were selectively cytotoxic *in vitro* to rat and human neurons and rat oligodendrocytes, independent of measured cytokine, immunoglobulin, or complement activation^[127,128]^. The observed effect was attributable, at least in part, to B cell-derived extracellular vesicles (EVs)^[129]^. EVs have been increasingly recognized for their importance in intercellular communication within both the healthy and diseased CNS^[130–133]^, and increased levels of EVs have been demonstrated in the CSF of MS patients^[134]^. EVs carry protein, lipid, and RNA cargo and could thus mediate toxicity in multiple ways. For instance, another *in vitro* study demonstrated that CSF from MS patients induced bioenergetic failure in rat neurons, again independent of levels of pro-inflammatory cytokines or other metabolites^[135]^. In this case, toxicity was mediated by elevated levels of certain ceramide species that are known to be enriched on the membranes of EVs^[136]^. It is also worth noting that, despite the emphasis above on toxic mediators, MS CSF and/or EVs may actually contain deficient levels of protective factors (i.e., an imbalance between injury and repair signals). Further study is warranted to better characterize the potential role of soluble CSF factors, including EVs, in the development of or protection from cortical damage in MS as well as the molecular pathways involved to identify potential novel therapeutic targets that may slow or prevent disease progression.

Another potential mechanism by which B cells may be involved in cortical injury relates to the description of EBV-infected plasma cells in both the MS meninges and parenchyma and their association with cytotoxic T cells in the perivascular cuffs of actively demyelinating lesions^[137]^. This suggests that EBV reactivation and the resulting antiviral response by CD8+ T cells within both the perivascular and meningeal compartments may propagate inflammation and mediate damage to the cortical tissue^[57,137]^. Additional mechanisms by which CD4+ and CD8+ T cells may injure neurons and oligodendrocytes have been described extensively in the context of WMLs, where both antigen-specific and antigen-independent mechanisms have been invoked (Reviewed^[138]^). It is reasonable to assume that similar mechanisms may take place in the cortex, albeit to a lesser extent given the relative paucity of parenchymal infiltration in cortical lesions.

Pro-inflammatory factors released into the CSF may also indirectly lead to cortical damage by activating CNS-resident cells, such as microglia and astrocytes^[88,109,112,139–141]^. It was previously shown that particular combinations of cytokines, defined by characteristic Th1, Th2, and monocyte/macrophage responses, greatly altered gene expression of MS relevant pathways in rat glial cultures^[139–141]^. Specifically, genes involved in antigen presentation, neuroprotection, axon-glial interactions, and metabolism were differentially regulated, suggesting that the activation state of nearby immune cells could impact the phenotype and likely also the function of glia in the MS CNS. Indeed, microglial activation is more robust in the cortex of individuals with more pronounced meningeal inflammation^[88,94]^, and these activated microglia are tightly associated with ongoing demyelination, axonal transections, and neuronal loss^[27,28,49]^. Microglia are themselves thought to propagate injury either directly by the release of pro-inflammatory cytokines^[125]^ or indirectly by exacerbating oxidative stress^[142–144]^, excitotoxicity^[125,145]^, and synaptic dysfunction^[125,145]^ in the surrounding cortex. Meanwhile, activated astrocytes may also promote CNS injury by influencing the recruitment and activation states of infiltrating and CNS-resident immune cells^[126,146,147]^ and, more directly, by reducing the crucial metabolic support available to surrounding neurons^[148]^. Injured astrocytes may also compromise the barrier function of the glia limitans and thus increase the susceptibility of the cortex to injury^[13,45]^. Furthermore, a certain population of pro-inflammatory astrocytes that was recently implicated in EAE-related CNS inflammation was also identified in the cortex of MS patients^[149]^. To what extent these or other populations of astrocytes drive cortical injury and progressive disease biology in MS patients is currently unknown, though in the context of EAE, it seems that activated astrocytes may be particularly pathogenic in later stages of disease^[146]^.

Overall, multiple mechanisms are likely to drive cortical injury in MS, and the heterogeneity of progression observed in patients both clinically and pathologically suggests that the predominant process(es) at play may differ across patients as well as within any given patient over time.

## DEMYELINATION *VS*. NEURODEGENERATION IN THE MS CORTEX

While axonal transections and neuronal loss are associated with demyelination in WMLs^[150]^, the relationship between demyelination and neuronal injury is less consistently described in the cortex of MS patients^[33,45,49,91,145,151]^. One possible explanation is that cortical neuronal loss results predominantly from the degeneration of axons injured in classic WMLs, rather than injury within the cortex itself. While this phenomenon has been shown to predominate in certain regions of the cortex, including leukocortical lesions^[15]^ and areas with extended axonal projections^[152,153]^, a growing body of literature now indicates that mechanisms driving neuronal injury in other areas of the cortex cannot be explained by retrograde degeneration alone^[15,69,154]^. In support of this, a recent post-mortem analysis defined a pathological subtype of MS, termed “myelocortical MS”, in which patients have typical spinal cord pathology and cortical injury in the absence of classic WMLs^[91]^. The substantial degree of cortical neuronal loss present in these patients suggests that cortical injury can occur independent of WMLs and that certain patients may be more or less susceptible to these different mechanisms.

The question then remains as to what additional pathogenic mechanisms are at play that culminate in the profound degree of cortical atrophy observed in MS patients over time. Demyelination itself may contribute to cortical neurodegeneration to a certain extent via loss of trophic support to axons^[155,156]^, reduced insulation from the local inflammation milieu^[49,156]^, and disruption of efficient signal transduction and energy production^[156,157]^, which together can result in axonal degeneration and cell death. However, several studies have failed to demonstrate a robust association between demyelination and neuronal/axonal loss in the cortex of MS patients^[33,45,91,151]^. While neuronal density was in fact reduced in demyelinated cortical lesions compared to control cortex, significant neuronal loss was also observed in the normal-appearing gray matter (NAGM)^[33,45]^. Though this finding indicates a dissociation between neurodegeneration and demyelination in the cortex, it also suggests there may exist a shared mechanism that contributes to cortical neurodegeneration more globally. Indeed, more pronounced meningeal inflammation is not only associated with subpial cortical lesions, but also with a gradient of microglial activation and neuronal loss in the NAGM^[44,45]^. It may be that the mechanisms by which meningeal inflammation drives subpial cortical injury, as described in the preceding sections, are also relevant to more diffuse cortical neuronal loss in MS, especially in the more superficial layers. It is interesting to note that several pathological differences between the superficial and deep layers of the cortex have been described in relation to neurodegeneration. Meningeal inflammation is generally associated with more superficial injury, while consequences of retrograde degeneration are seen in the deeper layers. Additionally, complement deposition is more extensive in cortical layers V and VI and is associated with greater neuronal loss, though notably independent of the extent of underlying WMLs^[158,159]^. Furthermore, differences in mitochondrial abnormalities can be seen with varying cortical depth^[160]^. Taken together, there are likely multiple processes that influence cortical neurodegeneration and the relative contribution of these processes may vary by cortical region (i.e., cortical lesions *vs*. NAGM, superficial *vs*. deep layers, *etc*.). It will be critical to disentangle exactly which mechanisms contribute to cortical demyelination and these different aspects of neurodegeneration in the future, as such discoveries may guide the development of distinct or perhaps complementary treatment approaches for progressive disease.

## IMPORTANCE OF IMMUNE-CNS CROSSTALK IN CORTICAL INJURY

Several lines of evidence support the concept that CNS-resident cells and infiltrating immune cells, whether contained within the perivascular spaces or meninges, communicate bidirectionally to both propagate CNS-compartmentalized inflammation and incite damage in the cortical parenchyma. Regarding the compartmentalized immune response, meningeal immune cell collections have been associated with more pronounced perivascular inflammation in the cortex, which may reflect greater meningeal inflammation solicited in response to more perivascular inflammation in the subjacent tissue, or alternatively, may indicate that more meningeal inflammation promotes greater immune cell perivascular infiltration in the underlying cortex via upregulation of certain chemokines and/or adhesion molecules on brain or endothelial cells^[57]^.

Next, with regard to cortical damage, B cells and other immune cells in the meninges (or in Virchow-Robin spaces) may produce factors that diffuse into the parenchyma and indirectly or directly induce cortical demyelination and neurodegeneration^[45,80,92,127,128,161]^. For example, secreted factors from immune cells may result in activated microglia that have been associated with ongoing demyelination and axonal injury in cortical lesions, most commonly at the lesion rim^[44,49,94]^, as well as in the NAGM^[44,45]^. Though microglial activation in response to a single insult may be generated as a compensatory response to acute CNS injury, it is thought that the homeostatic repair capacity of chronically activated microglia in the MS CNS is impaired^[125,162]^. Indeed, chronically activated microglia, as well as surrounding astrocytes, produce ROS and other toxic metabolites that lead to a number of metabolic derangements that impact neuronal viability^[13,49,125]^. For instance, ROS can directly damage neuronal and axonal mitochondria by inducing mitochondrial DNA (mtDNA) deletions and by reducing the expression of key enzymes in the respiratory chain complex^[160,163,164]^. Impaired and energetically inefficient mitochondria are then primed to produce more ROS, resulting in a deleterious feedforward mechanism^[165]^. Furthermore, the increased metabolic demand in the demyelinated axon, coupled with the surrounding inflammatory milieu, has been shown to amplify mtDNA deletions^[160,166]^ and further compounds the intracellular bioenergetic strain, resulting in cell death^[167]^. Activated microglia and astrocytes have also been shown to propagate excitotoxic damage in cortical neurons^[125,126,145]^. Specifically, in post-mortem tissue from progressive MS patients, activated microglia were associated with decreased expression of excitatory amino acid transporters (EEAT1 and EEAT2), which are critical for maintaining low extracellular glutamate concentration^[145]^. The loss of the protective mechanism was associated with increased axonal and synaptic damage in the cortex of progressive MS patients^[145]^. Taken together, microglial and astrocytic activation, which is increased in the brains of those with more pronounced meningeal inflammation, results in key changes in important aspects of neuronal metabolism that can perpetuate or perhaps incite neurodegeneration. It follows then that more pronounced meningeal inflammation is associated with increased microglial/astrocytic activation and more pronounced axonal injury^[21]^. However, it should be noted that certain metabolic changes, such as those associated with mitochondrial damage, have been observed in the cortex of MS patients without pronounced meningeal inflammation^[160]^, suggesting that other perhaps non-inflammatory mechanisms may contribute to progressive disease biology as well.

Nonetheless, the communication between meningeal immune collections and CNS cells is likely bidirectional in that activated cells within the CNS, including microglia, astrocytes, and perivascular infiltrates, signal back to both immune and stromal cells in the meninges to promote lymphocyte retention within the meningeal compartment^[104–106,168,169]^. Together, this complex network of cell-cell communication constitutes the dynamic CNS-compartmentalized immune responses that are likely central to disease progression.

## IMPLICATIONS FOR THERAPEUTIC INTERVENTION AND FUTURE DIRECTIONS

The literature often refers to relapsing MS disease mechanisms as “inflammatory” and progressive disease mechanisms as “degenerative”. However, the now well-documented association between meningeal inflammation, cortical injury, and clinical progression in MS points to underlying inflammatory mechanisms that may be viable targets for therapeutic intervention in progressive MS. This does not preclude an effort to further study as well as target degenerative disease mechanisms, but suggests that different types of inflammation, playing out in different anatomic compartments, contribute to distinct processes of CNS injury throughout the MS course.

With the recent approval of three drugs for the treatment of progressive forms of MS -ocrelizumab for PPMS and siponimod and cladribine for SPMS - it is of interest to consider the mechanisms by which these therapeutic benefits are mediated and, in particular, whether their ability to limit disease progression reflects, at least in part, targeting of CNS-compartmentalized immune responses relevant to cortical injury. A major challenge to establishing this is that each treatment has a robust effect on relapsing MS biology, which would be expected to limit relapse-related progression of disability. Here, we briefly consider how each agent may impact non-relapsing disease mechanisms.

Ocrelizumab, a humanized monoclonal antibody against CD20, was shown to have a modest but significant beneficial effect on limiting confirmed disability progression in both RRMS and PPMS patients^[170,171]^. Given the evidence supporting a pathogenic role of meningeal B cells in disease progression, it is interesting to speculate whether anti-CD20 therapy depletes these B cells in the CNS. While anti-CD20 therapy does reduce B cell counts in the CSF of MS patients^[172]^, it is not clear to what extent this reflects peripheral B cell depletion and hence less ongoing trafficking of B cells into the CNS *vs*. a direct effect of the anti-CD20 antibody on depletion of CNS B cells. There is likely only a small amount of the antibody that accesses the CNS and the extent to which the necessary killing machinery is present in the CNS (i.e., to enable complement-mediated and/or antibody-dependent cellular cytotoxicity of B cells) is unknown. The apparent persistence of CSF oligoclonal bands in the face of anti-CD20 therapy (at least over the initial two years of therapy) suggests that certain B cell populations, particularly plasma cells and plasmablasts, are highly resistant to such B cell depletion therapy^[173]^. Indeed, intrathecal delivery of anti-CD20 therapy in SPMS patients did not substantially reduce focal leptomeningeal enhancement, which was used as a proxy for the effect on discrete meningeal immune cell collections^[174]^. The apparent resistance of certain B cell populations to depletion in the CNS may be responsible for ongoing progression even in the absence of relapse in these patients^[175]^.

Siponimod, a sphingosine-1-phosphate receptor modulator, was approved for the treatment of active SPMS^[176]^. Siponimod, in addition to sequestering circulating immune cells in peripheral lymphoid structures, is expected to readily access the CNS where it may have a direct effect on CNS immune cells that express the S1P receptors, possibly contributing to CNS protection and/or repair, which to date has been demonstrated only in EAE^[177]^. A recent study using a T cell adoptive transfer model of EAE demonstrated that siponimod substantially reduced meningeal inflammation and subpial cortical injury via modulation of Th17 recruitment to and signaling within the CNS^[114]^. Notably, efficacy was highly dependent on the timing of drug initiation in that administration later in the disease course failed to prevent cortical damage^[114]^. This provides mechanistic insight into a plausible mechanism by which siponimod may exert beneficial effects on cortical pathology which, if true, would suggest that a greater effect on limiting progression may be achieved with earlier introduction of the therapy.

As with siponimod, the oral agent cladribine is approved for use in active SPMS patients^[178]^. Cladribine is a purine nucleoside analog that, when phosphorylated, accumulates in the nuclei of lymphocytes, resulting in DNA damage and eventually cell death^[179]^. Beyond selective depletion of lymphocytes, cladribine effectively penetrates the CNS where it could theoretically impact infiltrating and CNS-compartmentalized lymphocytes. Cladribine has also been shown to alter the inflammatory responses of microglia *in vitro*, such that they adopted a less activated phenotype^[180]^. Separately, in EAE models, intracerebroventricular administration of cladribine improved disease, in part, via a reduction in excitotoxicity^[181]^. While some studies have indicated that cladribine slows disability accumulation in both progressive and relapsing MS patients^[178,182,183]^, others have failed to show a statistically significant effect. Further studies are needed to better elucidate the ways in which cladribine may slow non-relapsing disease progression.

Efforts are ongoing to better understand how current and emerging therapies may beneficially impact both the CNS-compartmentalized immune response and neurodegeneration, wherein therapies could either interfere with detrimental cell processes or promote remyelination and repair of neural elements^[184,185]^. It is also possible that a combination of therapies addressing both inflammatory and de- or re-generative pathways may provide the greatest benefit. Of note, therapies directed at CNS-compartmentalized inflammation, including meningeal immune cell aggregates, would provide a critical biological proof-of-principle in progressive disease. The capacity to develop and translate such therapies into clinical practice rests, in part, on our ability to (1) improve upon existing imaging modalities to enhance the detection and monitoring of cortical lesions *in vivo*, and (2) identify key molecules and cell subsets in the meninges or parenchyma responsible for promoting and protecting against ongoing damage.

## Figures and Tables

**Table 1.** Studies investigating the association of cortical lesions with disease progression

Author	Method	Population(s) studied	Study design	Findings
*In vivo* imaging studies				
Calabrese *et al*.^[186]^, 2007	1.5T (DIR)	116 CIS, 163 RRMS, 101 SPMS 40 NC	Cross-sectional	Total number of CLs correlated with EDSS scores in all patient subgroups
Calabrese *et al*.^[101]^, 2009	1.5T (DIR)	70 RRMS	Cross-sectional	Total CL number and volume correlated with cognitive impairment in multiple domains as measured by the Rao’s Brief Repeatable Battery of Neuropsychological Tests. Total CL volume was an independent predictor of cognitive impairment
Calabrese *et al*.^[187]^, 2009	1.5T (DIR)	48 PPMS 22 NC	Longitudinal, 2 years	Total CL volume at baseline was an independent predictor of disability accumulation over the study period
Calabrese *et al*.^[90]^, 2009	1.5T (DIR)	48 Benign MS, 96 RRMS	Longitudinal, 1 year	Low number of CLs at baseline and minimal or absent accrual of both ICLs and LCLs over time were associated with benign MS compared to RRMS
Roosendaal *et al*.^[102]^, 2009	1.5T (DIR)	9 RRMS, 4 SPMS 7 NC	Longitudinal, 3 years	Total CL number at baseline predicted slower processing speed at follow-up. ICL and LCL volume at follow-up was associated with worse performance on tasks of processing speed and visuospatial memory
Calabrese *et al*.^[188]^, 2010	1.5T (DIR)	76 RRMS, 31 SPMS	Longitudinal, 3 years	Increases in CL number and volume were greatest in patients with clinical worsening compared to stable patients. Baseline total CL volume was an independent predictor of EDSS worsening over the study period in both RRMS and SPMS patients
Filippi *et al*.^[39]^, 2010	1.5T (DIR)	80 CIS (training) 39 CIS (validation)	Longitudinal, 4.6 years (training) 2.3 years (validation)	The presence of one or more ICLs on baseline MRI in CIS patients was an independent predictor of subsequent conversion to clinically definite MS
Bagnato *et al*.^[189]^, 2010	3T (3D IRSPGR)	17 RRMS, 4 SPMS 21 NC	Cross-sectional	Patients with CLs demonstrated worse performance on tests of delayed recall, but this relationship was not independent of WML volume
Mike *et al*.^[190]^, 2011	3T (3D IRSPGR, 3D FLAIR)	20 RRMS, 6 SPMS	Cross-sectional	Total CL number and volume correlated with worse performance on working memory as well as worse EDSS scores. Total CL number also correlated with worse performance on verbal learning and memory
Nelson *et al*.^[59]^, 2011	3T (DIR, PSIR)	39 MS, no subtype specified	Cross-sectional	The number of LCLs located predominantly in the cortex correlated with cognitive impairment, as did the number of these LCLs plus ICLs. ICLs alone did not correlate with cognitive impairment, though these lesions tend to be significantly smaller
Giorgio *et al*.^[43]^, 2011	1.5T (DIR)	15 RIS	Cross-sectional	Total CL number and volume correlated with WML volume, presence of OCBs, and DIT criteria on MRI and were present in all subjects classified as having a very high probability of conversion to clinically definite MS
Calabrese *et al*.^[37]^, 2012	1.5T (DIR)	157 RRMS, 35 Pediatric MS, 45 Benign MS, 44 PPMS, 31 SPMS	Longitudinal, 5 years	Patients with relapse independent EDSS progression had a greater number and volume of CLs at baseline, higher rates of CL accumulation, and a greater increase in CL volume. CL volume at baseline was an independent predictor of EDSS and cognitive worsening over the study period in all disease subtypes
Nielsen *et al*.^[70]^, 2013	7T ((FLASH)-T2*)	10 CIS, 8 RRMS, 9 SPMS	Cross-sectional	Numbers of total CLs, LCLs, and SPLs (but not ICLs) were significantly associated with EDSS scores, with SPLs demonstrating the strongest association. Numbers of total CLs and all CL subtypes were negatively correlated with at least one measure of cognitive performance. LCLs correlated with the greatest number of cognitive measures
Calabrese *et al*.^[40]^, 2013	1.5T (DIR)	334 RRMS (training) 88 RRMS (validation)	Longitudinal, 5 years	CL volume at baseline was an independent predictor of progression from RRMS to SPMS
Kolber *et al*.^[191]^, 2015	3T (3D DIR, 3D FLAIR)	29 CIS, 93 RRMS	Cross-sectional	Number of CLs correlated with worse performance on tests of working memory and semantic word fluency.
Harrison *et al*.^[69]^, 2015	7T (3D MPRAGE, 3D MPFLAIR)	30 RRMS & 6 PP/SPMS 15 NC	Cross-sectional	Patients with higher EDSS scores had a higher total CL number and volume as well as higher SPL volume. Total CL volume and LCL volume, in particular, were both independent predictors of cognitive impairment
Geisseler *et al*.^[192]^, 2016	1.5T (3D DIR, 3D MPRAGE, 3D FLAIR)	42 RRMS 43 NC	Cross-sectional	Patients with CLs demonstrated more pronounced cortical thinning and worse performance on memory tasks compared to patients without CLs and controls
Curti *et al*.^[193]^, 2018	3T (3D DIR)	47 RRMS, 11 SPMS, 2 PPMS	Cross-sectional	Total CL number correlated with poor performance in multiple cognitive domains
Scalfari *et al*.^[38]^, 2018	1.5T (3D DIR, 3D MPRAGE, 3D FLAIR)	219 RRMS	Longitudinal, 7.9 years	A greater number of CLs at baseline predicted a higher risk of conversion to SPMS and shorter time to conversion over the study period. Baseline CL volume was an independent predictor of progression to SPMS
Treaba *et al*.^[42]^, 2019	7T (T2*-weighted gradient-echo)	20 RRMS, 13 SPMS 10 NC	Longitudinal, 1.5 years	Total CL volume was an independent predictor of baseline EDSS and EDSS change at follow up
Post-mortem clinical-pathological correlation studies				
Magliozzi *et al*.^[44]^, 2007	Post-mortem histology	29 SPMS, 7 PPMS 3 NC	Clinical-pathological correlation	Patients with follicle-like structures in the meninges had more pronounced cortical (particularly subpial) demyelination and more severe clinical course, characterized by younger age at onset, irreversible disability, and death
Magliozzi *et al*.^[45]^, 2010	Post-mortem histology	37 SPMS 14 NC	Clinical-pathological correlation	In a larger SPMS cohort, patients with follicle-like structures in the meninges had more pronounced subpial cortical pathology and more severe clinical course, characterized by younger age at onset, age at death, age at wheelchair dependence, and time to wheelchair use
Howell *et al*.^[94]^, 2011	Post-mortem histology	123 SPMS 6 NC	Clinical-pathological correlation	Patients with follicle-like structures in the meninges had a greater extent of meningeal inflammation, cortical demyelination, and activated microglia in the cortex. Cases with follicle-like structures also had a more severe clinical course, characterized by younger age at onset, age at death, age at progression from relapsing to progressive disease, and age at wheelchair dependence
Choi *et al*.^[93]^, 2012	Post-mortem histology	26 PPMS 6 NC	Clinical-pathological correlation	More pronounced meningeal inflammation was associated with more severe cortical demyelination and clinical course, characterized by younger age at time of death, shorter disease duration, and shorter time from wheelchair use to death
Kooi *et al*.^[56]^, 2012	Post-mortem histology	1 PRMS, 11 SPMS, 23 PPMS, 6 Unclassified MS	Clinical-pathological correlation	A subset of patients with CLs had CLs with a rim of activated microglia. These patients had a younger age at time of death and shorter disease duration than patients with cortical pathology in the absence of rim-associated microglia or patients without extensive CLs
Magliozzi *et al*.^[57]^, 2013	Post-mortem histology	44 SPMS	Clinical-pathological correlation	Patients with follicle-like structures in the meninges had more pronounced cortical demyelination, perivascular inflammation in the cortex, and more severe clinical course. No differences in clinical severity were observed between patients with follicle-like meningeal inflammation with cortical perivascular infiltrates and those without perivascular inflammation
Bevan *et al*.^[88]^, 2018	Post-mortem histology	12 Acute MS, 18 SPMS, 3 PPMS, 11 NC, 6 OIND	Clinical-pathological correlation	Patients with acute MS (average disease duration of 2 years prior to death) exhibited significant cortical demyelination, the majority in a subpial pattern. Greater myeloid cell activation was observed in cases with more pronounced meningeal inflammation
Trapp *et al*.^[91]^, 2018	Post-mortem histology	12 MCMS, 12 TMS	Clinical-pathological correlation	MCMS were characterized by cortical and spinal cord pathology in the absence of cerebral WMLs. Age at death and EDSS did not differ between MCMS and TMS. Cortical demyelinated lesion area was similar between MCMS and TMS. MCMS had decreased cortical neuronal densities compared to controls despite lack of WMLs

CIS: clinically isolated syndrome; CLs: cortical lesions; DIR: double inversion recovery; DIT: dissemination in time; EDSS: expanded disability status scale; FLAIR: fluid-attenuated inversion recovery; FLASH: fast low angle shot; ICLs: intracortical lesions; IRSPGR: inversion-recovery spoiled gradient-recalled echo; LCLs: leukocortical lesions; MCMS: myelocortical MS; MPRAGE: magnetization-prepared rapid gradient echo; MRI: magnetic resonance imaging; NC: normal controls; OCBs: oligoclonal bands; OIND: other inflammatory neurologic disease; PPMS: primary progressive multiple sclerosis; PSIR: phase-sensitive inversion recovery; RIS: radiologically isolated syndrome; RRMS: relapse-remitting multiple sclerosis; SPLs: subpial lesions; SPMS: secondary progressive multiple sclerosis; TMS: typical MS; WMLs: white matter lesions
